# A large left atrial lipoma combined with coronary artery disease

**DOI:** 10.1186/s13019-017-0633-1

**Published:** 2017-08-22

**Authors:** Yun Liu, Xiaomei Zheng, Yu Du, Zhicheng Zhu, Tiance Wang, Rihao Xu, Dan Li, Kexiang Liu

**Affiliations:** The Second Affiliated Hospital of Jilin University, Changchun, China

**Keywords:** Lipoma, Cardiac benign tumor, Coronary artery bypass

## Abstract

**Background:**

Primary benign tumors of the heart are extremely rare and usually difficult to diagnose for their asymptomatic signs.

**Case presentation:**

A 66-year-old woman was admitted for shortness of breath caused by a large left atrial lipoma combined with coronary artery disease. Next, we successfully performed simultaneous curative surgery for the large cardiac lipoma and coronary artery bypass grafting with a “starfish” and no cardiopulmonary bypass was used.The patient was discharged on the eighth postoperative day in a good condition, and has remained asymptomatic at the 5-month follow-up.

**Conclusions:**

Lipomas are rare and difficult to diagnose, while computed tomography and computed tomography angiography can give us very important clues. Surgery is necessary. We can introduce a “starfish”to the operationand the cardiopulmonary bypass is unnecessary for the left lipoma with coronary artery disease.

## Background

Cardiac lipomas are extremely rare, accounting for 8.4% of all primary tumors [1]and found at a frequency of only 0.001%–0.28% at autopsy [2]. They are frequently located in the left ventricle or right atrium. Left artrial lipomas are very rare with only 3 references in all of the epicardiac lipomas [3]. Clinically, this tumor is asymptomatic and found incidentally in the vast majority of cases [4]. Only large left atrial lipomas could alter atrial and ventricular functions and result in dyspnea, such as in our case.

## Case presentation

**Fig. 1:**
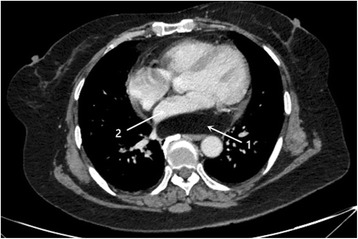
CTA showed that a large low density mass located on the posterior wall of the left atrium. The left atrium was compressed. The tumor was not enhanced on CTA (1. Tumor,2. Left atrium)

A 66-year-old female was admitted because of dyspnea. On admission, the temperature was normal, the pulse was 67 /min, the respiration rate was 20/min and the blood pressure was 108/68 mmHg. Laboratory examinations were normal. Computed tomography (CT) showed that a large low density mass located behind the posterior wall of the left atrium. The left atrium was compressed. The mass showed a density similar to adipose tissue and was not enhanced on computed tomography angiography(CTA) (Fig. 1). We highly suspected that it was a lipoma on the basis of its CT manifestations. The ejection fraction was 62%. The electrocardiogram was normal. Considering of her age, the coronary angiography was performed and the result revealed a 70% stenosis in the left anterior descending (LAD) branch. And we decided to perform simultaneous curative surgery for the cardiac lipoma and coronary artery bypass grafting (CABG).

**Fig. 2:**
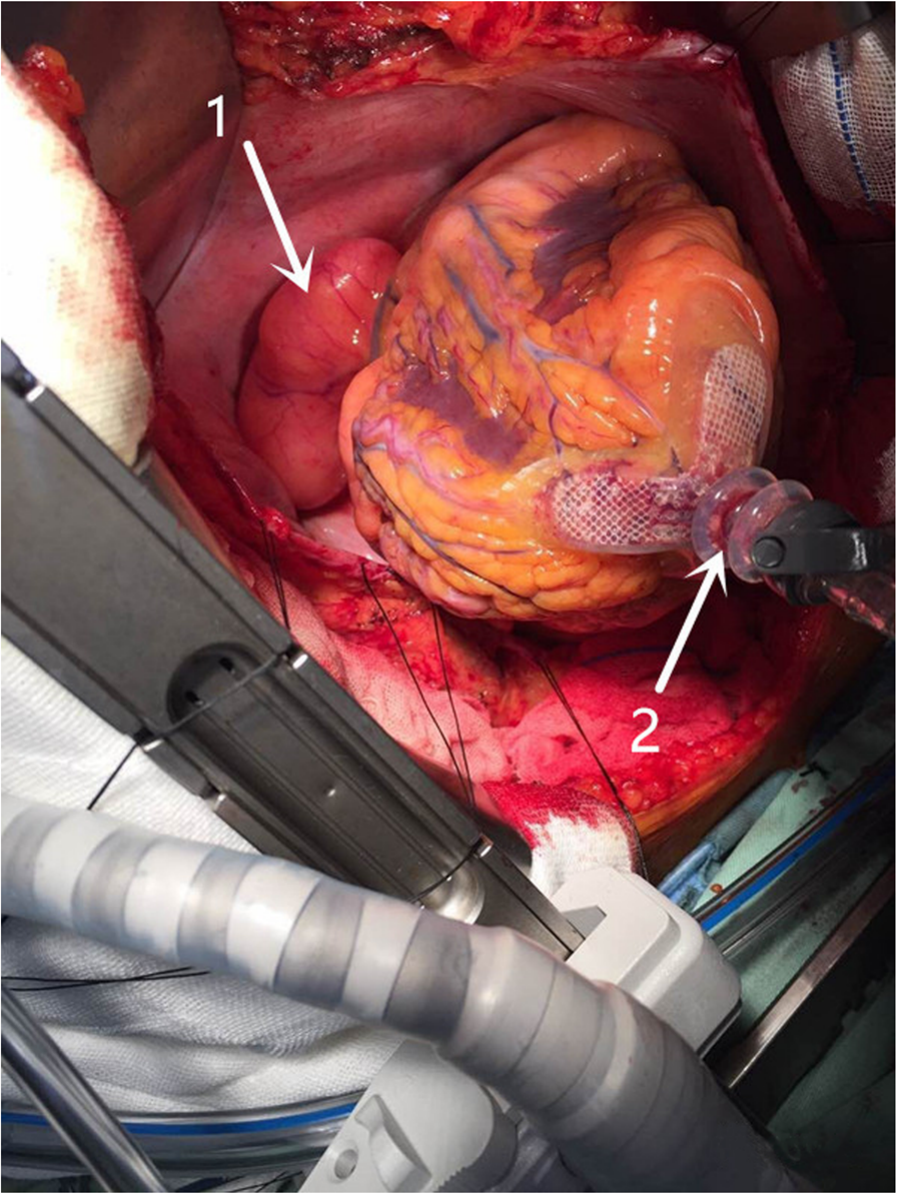
Intraoperative view of the tumor. A large lipoma was seen at thoracotomy with a starfish.(1.lipoma; 2.starfish)

**Fig. 3:**
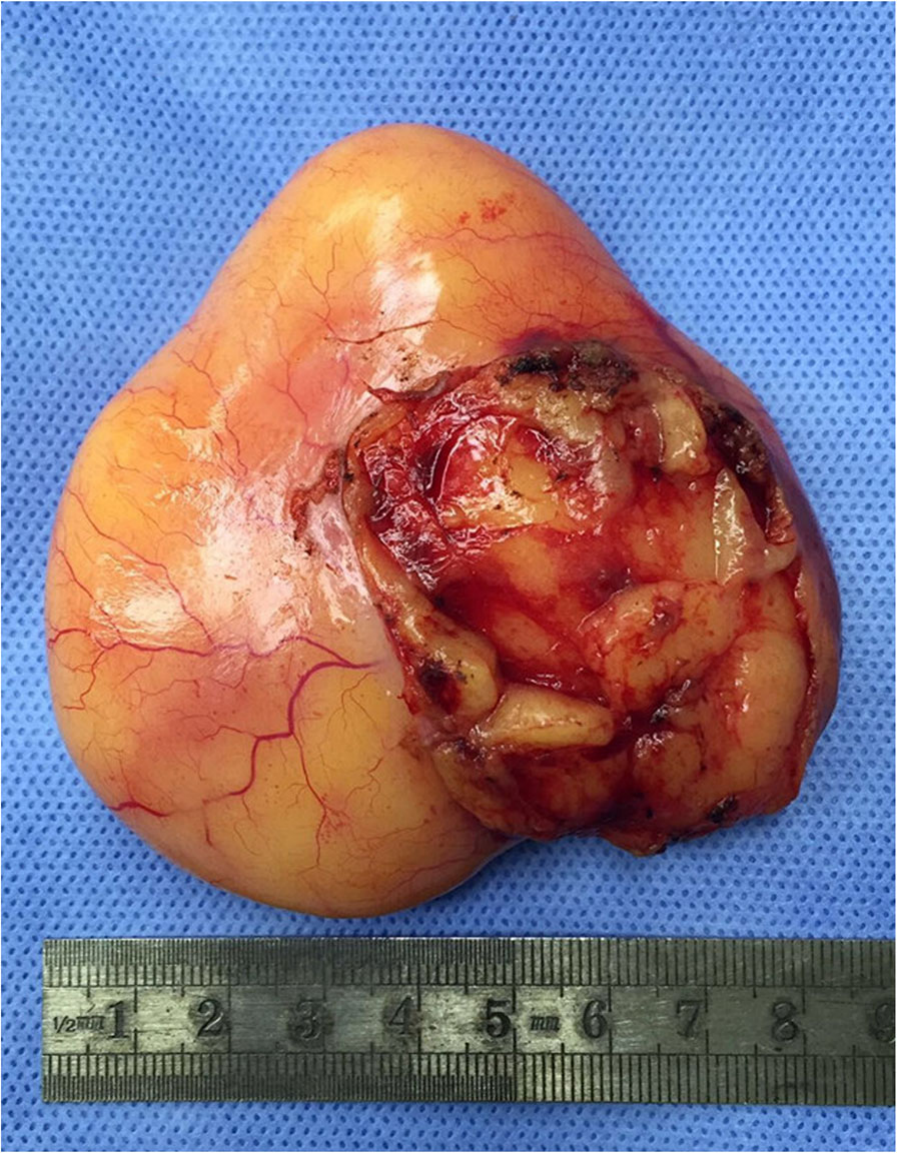
The mass was attached to the posterior pericardium beneath the left pulmonary artery with a stalk connected to the left atrium

A median sternotomy and pericardial incision were performed undergeneral anesthesia. After we lifted up the heart with a “starfish”, a soft, yellow tumor which originated from the left atrium without any invasion to the pericardium was found (Fig. 2). Then we excised the basal part of the tumor carefully with an electrome and no cardiopulmonary bypass was used. The mass was removed completely and measured about 8 cm × 8 cm × 4 cm (Fig. 3). Next, we performed the anastomosis between the anterior descending branch and the left internal mammary artery. The procedure was smooth, and postoperative recovery was good.

**Fig. 4:**
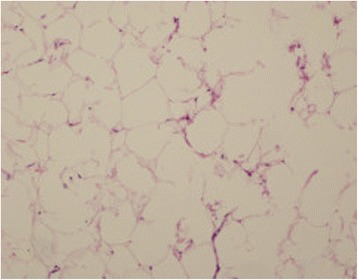
The histopathological examination revealed mature adipocytes

The histopathological examination revealed mature adipocytes confirming our suspicion of a limopa (Fig. 4). The patient was discharged on the eighth postoperative day in a good condition, and has remained asymptomatic at the 5-month follow-up. Echocardiography (ECHO) detected no signs of recurrence.

## Discussion

It’s usually difficult to diagnosis left arterial lipomas for its asymptomatic and low incidence. However, CT/CTA can give us very important clues to the diagnosis. When the CT showed a fat-like low density mass under the epicardium and was not enhanced on CTA, we should consider it as a lipoma first. Cardiac lipomas are benign and slowly growing, but for large lipomas, surgical resection is necessary to prevent tumor compression syndromes.

As we know, in all of the three cases, cardiac surgeons in one case performed the resection of the tumor and repaired left atrium(LA) surface with an autologous pericardial patch under cardiopulmonary bypass. In the other two cases, cardiac surgeons removed the tumor directly without cardiopulmonary bypass. In this case, we used a “starfish” to lift up the heart, which made the lipoma exposed well and removed easily. Also we underwent the off-pump coronary aortic bypass grafting (OPCABG). The result was good. So we consider that it’s unnecessary to perform the surgery under cardiopulmonary bypass for the left atrial lipoma pateints with coronary artery diseases.

## Conclusion

Lipomas are rare and difficult to diagnose, while computed tomography and computed tomography angiography can give us very important clues. Surgery is necessary. We can introduce a “starfish” to the operation and the cardiopulmonary bypass is unnecessary for the left lipoma with coronary artery disease.

## Abbreviations

CABG: Coronary artery bypass grafting
CPB: Cardiopulmonary bypass
CT: Computed tomography
CTA: Computed tomography angiography
ECHO: Echocardiography
LA: Left atrium
LAD: Left anterior descending
OPCABG: Off-pump coronary aortic bypass grafting
